# The Effect of the Concurrent Use of Angiotensin-Converting Enzyme Inhibitors or Receptor Blockers on Toxicity and Outcomes in Patients Treated with Radiotherapy: A Systematic Review and Meta-Analysis

**DOI:** 10.3390/ph18010105

**Published:** 2025-01-16

**Authors:** Wan-Chuen Liao, Hala Shokr, Corinne Faivre-Finn, Clare Dempsey, Kaye Janine Williams, Li-Chia Chen

**Affiliations:** 1 Division of Pharmacy and Optometry, School of Health Sciences, Faculty of Biology, Medicine and Health, The University of Manchester, Manchester Academic Health Science Centre, Manchester M13 9PT, UK; d08450001@ntu.edu.tw (W.-C.L.); kaye.williams@manchester.ac.uk (K.J.W.); li-chia.chen@manchester.ac.uk (L.-C.C.); 2 School of Dentistry, College of Medicine, National Taiwan University, Taipei 10048, Taiwan; 3 Division of Cancer Sciences, Faculty of Biology, Medicine and Health, The University of Manchester, Manchester M13 9PL, UK; corinne.finn@nhs.net (C.F.-F.); clare.dempsey@manchester.ac.uk (C.D.); 4 The Christie NHS Foundation Trust, Manchester M20 4BX, UK

**Keywords:** radiotherapy, polypharmacy, radiotherapy–drug interaction, angiotensin-converting enzyme inhibitors, angiotensin receptor blockers, radiotherapy-related adverse effects, cancer survival outcome

## Abstract

**Background/Objectives**: ACEIs protect against radiation pneumonitis by reducing angiotensin II production, oxidative stress, and inflammation. This study highlights the significance of concurrent angiotensin-converting enzyme inhibitor (ACEI) or angiotensin receptor blocker (ARB) use in radiotherapy by evaluating its impact on radiotherapy-related side effects and survival outcomes, addressing the gap in existing research and providing insights to guide clinical practice in oncology. **Methods**: The literature was retrieved from the MEDLINE, EMBASE, Web of Science, and Scopus databases from January 2000 to October 2024. Studies on adults (≥18 years) with histologically confirmed cancer, receiving ACEIs or ARBs during radiotherapy, were included. Radiotherapy-related side effects and clinical outcomes were analysed using odds ratios (ORs) and 95% confidence intervals (95%CIs), comparing ACEI/ARB users to non-users. Differences in the median survival time, recurrence, and death rates were also calculated. **Results**: Sixteen studies (14 cohort studies and two randomised trials) were included. ACEI users exhibited a 50% reduction in the risk of ≥grade 2 radiation pneumonitis (OR: 0.50, 95%CI: 0.32–0.77) in lung cancer and significant reductions in the odds of proctitis (80%, OR: 0.20, 95%CI: 0.12–0.33), haematuria (75%, OR: 0.25, 95%CI: 0.16–0.41), and rectal bleeding (61%, OR: 0.39, 95%CI: 0.30–0.51) in prostate cancer. ACEI/ARB users showed reduced symptomatic radiation necrosis in brain metastases and better 6-month functional independence in supratentorial glioblastoma. Among six studies reporting survival, ACEI/ARB users had longer median survival in early-stage non-small-cell lung cancer and glioblastoma but shorter survival in small cell lung cancer and brain metastases. ARB users had inconsistent survival rates for lung cancer. The varying survival outcomes suggest that ACEIs/ARBs have different effects depending on the cancer type and stage, potentially influenced by cancer-specific factors, treatment protocols, or disease progression. **Conclusions**: ACEI use is associated with a reduction in radiation pneumonitis, but evidence for other radiotherapy-related toxicity and survival outcomes remains inconsistent across cancer types and severities. Further research should carefully control for confounders.

## 1. Introduction

Radiotherapy, a standard cancer treatment, is often used alone or in conjunction with surgery, chemotherapy, or immunotherapy, comprising three main types: external beam radiation therapy, brachytherapy, and systemic radioisotope therapy [1]. Over 50% of patients with cancer receive radiotherapy for curative or palliative purposes [2]. However, radiotherapy toxicity can impact patients’ quality of life and treatment effectiveness [3,4,5,6,7]. Exploring strategies to support patients’ conditions is paramount in clinical oncology.

Patients with cancer often have multiple comorbidities, leading to high polypharmacy prevalence [8,9]. Optimising concurrent medication use during radiotherapy remains challenging. For example, hypertension, a major risk factor for cardiovascular disease and premature death globally [10], is commonly managed with angiotensin-converting enzyme inhibitors (ACEIs) and angiotensin receptor blockers (ARBs) [11,12]. Despite their widespread use, the effects of ACEIs and ARBs on radiotherapy toxicity and cancer outcomes are under-researched and poorly understood in real-world oncology practice. The lack of data raises questions about the safety and efficacy of ACEIs and ARBs in patients undergoing radiation therapy.

Pharmacologically, ACEIs and ARBs may mitigate radiotherapy-related side effects [13], but their clinical impacts remain inconclusive. Some retrospective cohort studies suggested that ACEIs and ARBs reduced radiation pneumonitis [14,15] and radiation-related proctitis [16,17]. Conversely, other retrospective cohort studies found that ACEIs did not consistently reduce symptomatic radiation pneumonitis [18,19], and ARBs have limited effects on radiation-related lung damage [20]. ACEIs and ARBs influence radiation pneumonitis through distinct mechanisms. ACEIs reduce angiotensin II (Ag-II) production and lower oxidative stress and inflammation, protecting against radiation pneumonitis [21,22]. ARBs block angiotensin type 1 (AT1) receptors but may enhance angiotensin type 2 (AT2) receptor activity, potentially exacerbating pneumonitis [23]. A systematic review and meta-analysis by Sun et al. (2018) [3] indicated that ACEIs significantly reduced symptomatic radiation pneumonitis in lung cancer, whilst ARBs did not show a significant effect [3]. However, this review only focused on patients with ≥grade 2 pneumonitis within 12 months post-radiotherapy and comprised seven studies.

Conflicting results regarding the survival outcomes with ACEIs or ARBs in patients with cancer have also been noted. Some retrospective cohort studies have reported improved survival outcomes [24,25,26], whilst others have found no significant association between these drugs and the survival of patients with primary glioblastoma undergoing chemotherapy or radiotherapy [27]. These discrepancies may stem from variations in the cancer severity, stage, or type of combined therapies.

Given the lack of consensus and conflicting data on the safety and efficacy of ACEIs and ARBs during radiation therapy, this review provides an updated analysis by including recent studies on radiation pneumonitis. Whilst data on additional toxicity types and radiotherapy sites remain limited, our synthesis offers timely insights and addresses critical gaps, supporting the need for clearer clinical guidelines in oncology. This systematic review and meta-analysis aimed to evaluate the impact of ACEIs and ARBs on radiotherapy-related side effects and survival outcomes in patients with cancer receiving radiotherapy to inform routine clinical practice.

## 2. Materials and Methods

This systematic review and meta-analysis followed the Preferred Reporting Items for Systematic Reviews and Meta-Analyses (PRISMA) statement guidelines (Supplementary Material Table S1) [28]. The protocol has been registered at PROSPERO (no. CRD42023487336).

### 2.1. Selection Criteria

This study’s inclusion and exclusion criteria are summarised as follows (Table 1).

### 2.2. Types of Studies

Original articles about prospective or retrospective cohort studies, cross-sectional studies, and clinical trials were included. Case–control studies, case series, case reports, systematic reviews, meta-analyses, conference abstracts, editorials, letters to editors, commentaries, and grey literature were excluded.

### 2.3. Types of Participants

The study included participants who were patients aged 18 and above with histologically confirmed cancer and scheduled for various doses and regimens of radiotherapy. Exclusions were those under 18, studies with mixed age groups, patients receiving neoadjuvant radiotherapy or diagnostic radiology, and patients with cancer types not amenable to radiotherapy.

### 2.4. Types of Interventions

Patients who received an oral dose of ACEIs or ARBs, alone or combined with other medications, during radiotherapy treatment were included. Patients not concurrently using or those receiving intravenous therapy of ACEIs or ARBs were excluded.

### 2.5. Types of Outcome Measures

This study considered two outcome measures: radiotherapy-related side effects and survival outcomes. Radiotherapy-related side effects occurring during or immediately after radiotherapy were included. Survival outcomes were assessed through survival or recurrence results. Radiation-related toxicity occurring before ACEI or ARB administration was excluded.

### 2.6. Data Sources and Search Strategies

A comprehensive search was conducted across electronic databases, including MEDLINE, EMBASE, Web of Science, and Scopus, from 1 January 2000 to 21 October 2024. The timeframe was chosen based on initial searches, which indicated that the relevant literature emerged after 2000, and the final version of this review was conducted in October 2024. Structured search strategies (File S1) used controlled vocabulary and keywords aligned with the inclusion and exclusion criteria (Table 1). Search restrictions were applied, including English language and human studies.

### 2.7. Study Selection

Two reviewers (WCL and HS) screened the titles and abstracts independently of articles retrieved from the electronic databases search (Table 1) using a pre-designed electronic screening form. The consistency between the two reviewers was assessed by the intraclass correlation coefficient (two-way mixed-effect model with absolute agreement) [29]. Any discrepancies were resolved through reviewer discussion and, if needed, with a third reviewer (LCC) to reach a consensus. The full texts of potentially eligible articles underwent independent review by two reviewers (WCL and HS) to finalise the study selection.

### 2.8. Data Extraction and Management

Two reviewers (WCL and HS) independently extracted data from each study using a standardised electronic data extraction sheet. Discrepancies were resolved by a third reviewer (LCC). Extracted data included the study information (title, leading author, country, year of publication), study design, setting, targeted population (disease and cancer stages), intervention (ACEI or ARB exposure), comparison, outcome measures, and follow-up period. Study results were collected, including the proportion (or the number of the numerator and denominator) of adverse events occurring during or immediately after radiotherapy and the number or proportions of survival outcomes. All radiotherapy-related side effects reported in studies meeting the inclusion criteria were extracted for analysis in this review. If raw data were unavailable, the risk ratio, hazard ratio, mean (with standard deviation), median (with range) of survival duration, or other results were converted into raw data.

### 2.9. Risk of Bias Assessment

A quality assessment of all included studies was conducted using the Cochrane Risk of Bias Assessment Tool (RoB 2) for randomised controlled trials and the Risk of Bias in Non-Randomised Studies of Interventions Tool (ROBINS-I) for non-randomised studies [30,31]. The results of the assessment categories were tabulated for clarity.

### 2.10. Data Analysis and Presentation

All outcomes were compared between the exposed group (ACEI, ARB, or ACEI/ARB users) and the non-exposed (non-users) group. The ACEI/ARB group comprised patients taking either or both of these drugs without explicit differentiation in the study. The proportions of radiotherapy-related side effects, categorised by different organ systems, were synthesised using a random effect model (DerSimonian and Laird method) [32]. The random-effects model was selected for the meta-analysis to account for variability between the studies due to differences in the methodology and settings, ensuring robust and generalisable pooled estimates. Pooled effect sizes were reported using odds ratios (ORs) and 95% confidence intervals (95%CIs), with heterogeneity assessed using the *I*^2^ test (%). Survival outcomes, including the difference in median survival time (by subtraction) and ORs of survival rates, were calculated and synthesised where appropriate. Heterogeneity among studies was addressed through subgroup analyses, such as the classification of ACEIs and ARBs into different categories. The meta-analysis was conducted using STATA (Release 14. College Station, TX, USA: StataCorp LLC).

## 3. Results

### 3.1. Selection of Studies

**Figure 1:**
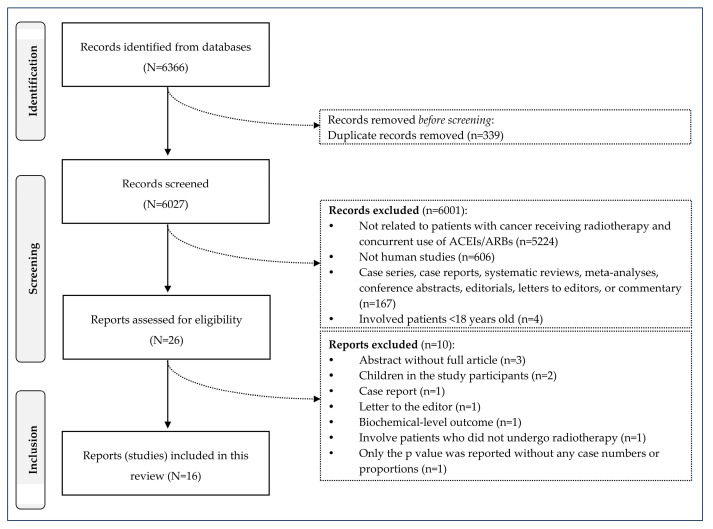
Selection of studies. (Note) ACEIs: angiotensin-converting enzyme inhibitors. ARBs: angiotensin receptor blockers.

Of 6366 records identified from the electronic database searches, 339 duplicates and 6001 irrelevant records were removed. After full-text screening, ten studies were excluded, leaving 16 studies (4576 patients) for analysis (Figure 1). The intraclass correlation coefficient between the two reviewers was 0.882 (95%CI: 0.877, 0.887), indicating good consistency.

### 3.2. Characteristics of Studies

The review included 16 studies: 14 cohort studies and two randomised trials conducted on patients with various cancers, including lung cancer (n = 11) [14,15,18,19,20,21,23,26,33,34,35], prostate cancer (n = 2) [16,17], brain metastases (n = 1) [36], glioblastoma (n = 1) [37], and pelvic malignancies (n = 1) [38] (Table 2). Most studies (n = 12) compared ACEI users and non-users [14,16,17,18,19,20,21,23,33,34,35,38], followed by comparing ACEI/ARB users and non-users (n = 8) [14,15,19,20,21,33,36,37], and only four studies compared ARB users and non-users [14,19,20,26].

### 3.3. Quality Assessment

In the risk of bias assessment, the two authors demonstrated consistency and agreement. The two included randomised trials exhibited limitations, with one study failing to report the follow-up period [34] and the other analysing only 61% of the randomised patients (n = 33) [35] (Table S2). Most non-randomised studies showed a moderate risk of bias by including patients with different cancer stages that could impact the outcomes (n = 12) [15,16,17,18,19,20,23,26,33,36,37,38], lacking specification of the follow-up period (n = 4) [23,33,37,38], and substantial loss to follow-up (31%) at the 1-year mark (n = 1) [38]. Additionally, two studies were considered to have a serious risk of bias, one for reporting only *p* values, without exact case numbers or proportions in each group [23], and the other for exclusively including male patients with lung cancer undergoing radiotherapy [33] (Table S3).

### 3.4. Radiation Pneumonitis

The pooled effect size results of seven studies [14,18,19,20,21,33,35] showed that ACEI use was associated with a significantly reduced risk of ≥grade 2 radiation pneumonitis (OR: 0.50, 95%CI: 0.32, 0.77; *I*^2^: 10%) than in non-users. This indicates a 50% reduction in the risk of radiation pneumonitis in ACEI users. The low *I*^2^ value (10%) suggests minimal heterogeneity, indicating robust results. However, the pooled results from three [14,19,26] and six studies [14,15,19,20,21,33] indicated no significant difference in the risk of developing ≥ grade 2 radiation pneumonitis when comparing ARB (OR: 1.15, 95%CI: 0.29, 4.64; *I*^2^: 56%) or ACEI/ARB users (OR: 0.83, 95%CI: 0.45, 1.51; *I*^2^: 63%) with non-users, respectively. The higher *I*^2^ values (56% and 63%) suggest moderate to substantial heterogeneity, indicating that these results are less consistent across studies (Table 3).

Likewise, despite wide confidence intervals, the pooled effect size results from three [14,21,33] and five [14,18,20,21,35] studies showed that ACEI users significantly increased the freedom from symptomatic radiation pneumonitis rate compared to non-users at six months (OR: 4.78, 95%CI: 1.44, 15.84; *I*^2^ < 0.1%) and 12 months (OR: 2.16, 95%CI: 1.13, 4.15; *I*^2^: 19%), respectively. This indicated that ACEI users were 4.8 times more likely to remain free of symptomatic radiation pneumonitis at six months and 2.16 times more likely at 12 months. The results from a single study on non-small-cell lung cancer (NSCLC) indicated no significant difference in the freedom from symptomatic radiation pneumonitis rate between ARB users and non-users [14]. ACEI/ARB users significantly reduced the risk at six months (OR: 10.98, 95%CI: 1.41, 85.38) and 12 months (OR: 3.47, 95%CI: 1.12, 10.76) compared to non-users, with a wide confidence interval (Table 4).

### 3.5. Other Adverse Events and Results

Other radiotherapy-related side effects reported in a single study were not synthesised (Table 5). Chowdhary et al. (2018) found that ACEI/ARB users had a significant reduction in the risk of symptomatic radiation necrosis (OR: 0.13, 95%CI: 0.03, 0.58) and the one-year symptomatic radiation necrosis rate (OR: 0.10, 95%CI: 0.01, 0.74) compared with non-users among patients with brain metastases [36].

Januel et al. (2015) discovered no significant difference in the 1-month functional independence rates but noted a significantly improved 6-month functional independence rate in ACEI/ARB users compared to non-users among patients with supratentorial glioblastoma [37]. Two studies showed that patients with prostate cancer receiving ACEIs had significantly lowered risks of proctitis (OR: 0.20, 95%CI: 0.12, 0.33) [16], developing haematuria (OR: 0.25, 95%CI: 0.16, 0.41) [17], and rectal bleeding (OR: 0.39, 95%CI: 0.30, 0.51) [17] compared to non-users.

Additionally, patients with NSCLC receiving ACEIs showed no significant difference in acute kidney injury and hypotension compared to non-users [34]. Patients with early-stage lung cancer receiving ARBs showed no significant difference in the risk of pulmonary fibrosis compared to non-users [26].

Sio et al. (2019) reported that ACEI users showed less acute pulmonary distress (score: 77.5 vs. 42, where a higher score suggests less distress) in patients with NSCLC [34]. Wedlake et al. (2012) found that ACEI users presented attenuated acute inflammatory bowel disease (asymptomatic score: 58.8 vs. 55.8) among patients with pelvic malignancies compared to non-users [38].

### 3.6. Survival Outcomes

In patients with early-stage NSCLC [23,26] and glioblastoma [37], it was indicated that the median survival time of ACEI/ARB users was longer than that of non-users (difference: 3 to 53.4 months). Conversely, it was shorter than that of non-users in patients with small-cell lung cancer [33] and brain metastases [36] (Table S4).

Harder et al. (2015) [20] reported no significant difference in the two-year overall survival rate of patients with primary lung cancer between ACEI users and non-users. However, ARB users showed a significantly worse result (OR: 0.44, 95%CI: 0.21, 0.93) than non-users. Conversely, Maloney et al. (2022) [26] found that patients with early-stage lung cancer receiving ARBs had no significant difference in the total recurrence rate but a significantly lower total death rate (OR: 0.21, 95%CI: 0.08, 0.58) when compared to non-users. Wedlake et al. (2012) [38] found ACEI users had no significant difference in the risk of death due to disease progression rate or the cancer-related treatment toxicity rate compared to non-users among patients with pelvic malignancies.

## 4. Discussion

This review assessed the impact of concurrent ACEI/ARB usage in patients with cancer undergoing radiotherapy, with a primary focus on radiotherapy-related toxicity. Clinicians may anticipate that ACEI use is robustly associated with a significantly reduced risk of ≥grade 2 radiation pneumonitis. In early-stage lung cancer, NSCLC and glioblastoma, ACEI/ARB use was associated with longer median survival times compared to non-use. However, survival rate data are limited and inconclusive.

Radiation interacts with biological systems by influencing tissues and cellular processes, with the effects varying based on the frequency, power, and exposure duration. It can induce oxidative stress, DNA fragmentation, and neurotransmitter signal delays but also has neutral or beneficial applications, such as in cancer treatment [39]. Radiation pneumonitis is a serious complication that could cause significant morbidity, restrict radiotherapy treatment dosages, and increase mortality [40]. ACEIs and ARBs have different pharmacological actions, which might influence the radiation pneumonitis outcomes. ACEIs inhibit the conversion of angiotensin I (Ag-I) to II and reduce the activation of AT1 and AT2 receptors, whilst ARBs selectively block AT1 receptors by antagonising Ag-II binding to AT1 receptors. ACEIs have been shown to protect against radiation-induced oxidative stress and pneumonitis by reducing inflammatory reactive oxygen species in animal [22] and clinical observation [21] studies. In contrast, ARBs might exacerbate radiation pneumonitis [23] by blocking AT1 receptors and enhancing AT2 receptor stimulation. ACEIs and ARBs are thought to inhibit fibrosis through renin–angiotensin system receptors [13], particularly the AT1 receptor, linked to transforming growth factor-β1 release, which is associated with radiation-related fibrosis [41,42,43,44].

Consistent with the previous literature indicating ACEIs’ effectiveness in preventing lung injury, whilst the efficacy of ARBs remains controversial [3,14,45], our review found ARBs’ impacts on the radiation pneumonitis (n = 3) and pulmonary fibrosis rates (n = 1) to be inconclusive due to the limited studies identified. Furthermore, the constrained mitigation of radiotherapy’s side effects by ACEIs/ARBs might be due to factors such as radiation not being the primary stimulus to induce transforming growth factor-β production [18] or hindrances to their effectiveness, such as large treatment volumes, less accurate radiation delivery [21], or inadequate drug dosages [18].

In addition, other potential confounding factors may impact radiotherapy-related toxicity and cancer survival, including patient characteristics (age, gender, race, smoking status, alcohol consumption), comorbidities, tumour characteristics (type and stage of cancer), medication use (statins, non-steroidal anti-inflammatory drugs, and corticosteroids), radiotherapy-related variables (type, dose, and radiation dosimetry factors), and the follow-up duration [14,19,46]. Two of the included studies used propensity score matching [16] and the genetic propensity method [17], considering comorbidities, health behaviours, and other variables to balance the ACEI/ARB users and non-users. However, potential biases remained due to unmeasured confounders in retrospective studies.

This review found that the beneficial impact on adverse events appeared more pronounced than that on the survival outcomes. Hypertensive patients with early-stage lung cancer may safely continue ACEIs during radiotherapy without concerns. However, due to a lack of evidence, it is unclear whether non-hypertensive patients should be prescribed ACEIs/ARBs specifically for the prevention of radiation pneumonitis.

This review also identified studies reporting ACEIs/ARBs’ impacts on radiotherapy-related complications in other organ systems, such as reduced symptomatic radiation necrosis in patients with brain metastases [36]. This effect may be attributed to the inhibition of the Ag-II peptide, which modulates the oxidative damage pathway [36]. The concurrent use of ACEIs/ARBs with radiotherapy may improve the clinical outcomes and functional independence in patients with supratentorial glioblastoma [37]. Similarly, ACEIs/ARBs have been proven to prevent perirhinal cortex-dependent cognitive function damage and alleviate chronic cognitive impairment in irradiated animal models [47,48]. The reduction in proctitis by ACEIs may be related to enzyme inhibition or the antioxidant properties of ACEIs, although the exact mechanism remains unclear [16]. The protective effect of ACEIs in patients with prostate cancer has been suggested to possibly be linked to increased levels of renin and Ag-I, with subsequent downstream effects on bradykinin and prostaglandins, whilst potentially reducing Ag-II. However, further investigation may be required to determine whether the clinical effects are mediated through these pathways or involve a mechanism independent of these receptor interactions [17].

Furthermore, ACEI/ARB users had longer median survival times than non-users in early-stage NSCLC and glioblastoma. However, a definitive conclusion cannot be established due to the limited number of studies and contrasting results. Factors such as the cancer type [49], follow-up duration [50], and ACEI or ARB usage [49] may influence the survival outcomes, leading to inconsistencies across studies.

Compared to past studies primarily focused on illustrating potential cell mechanisms in vitro or providing reviews [51,52], this study synthesised the current evidence on the association between the use of ACEIs/ARBs and radiotherapy-related side effects, as well as survival outcomes, across various types of cancer. Moreover, unlike previous meta-analyses [3] and most existing literature, this review went beyond radiation pneumonitis to provide a structured, systematic review and meta-analysis of human studies, offering valuable and practical insights for clinical oncology.

However, several limitations must be acknowledged. Including retrospective observational studies may have introduced potential bias due to confounding factors, and variations in cancer stages could impact survival outcomes or obscure the effects of ACEIs/ARBs [19]. Retrospective studies also encounter challenges in accurately assessing medication adherence and durations of use, whilst some randomised trials fail to achieve sufficient statistical power [34,35]. Additionally, variations in radiotherapy protocols and differences in ACEI/ARB dosages across studies may further influence the observed outcomes. Limiting the search to English language sources may have introduced bias. Additionally, the primary focus of the included studies was not on the effects of ACEIs/ARBs but on radiotherapy and cancer treatments, resulting in more indirect findings. The wide 95%CIs for some ORs indicate potential uncertainty, requiring the cautious interpretation of the results.

Given the limited research on ACEIs/ARBs’ effects in radiotherapy, we included all eligible sources. Notably, we incorporated at least two additional articles from 2022 and 2023 on radiation pneumonitis, distinguishing our analysis from previous work. Whilst we aimed to broaden the scope by examining additional toxicity types and radiotherapy sites, data on these outcomes were generally limited to one or two studies per category. Nonetheless, we believe that our systematic review and meta-analysis provides a timely and clinically valuable synthesis of the current knowledge on ACEI/ARB use in radiotherapy.

Although randomised controlled trials are the gold standard in establishing causality in clinical research, the cost, ethics, and participant recruitment pose significant challenges [53]. Instead, well-designed observational studies should be encouraged, employing appropriate methodologies to manage confounders, reduce the risk of biases, and infer associations. Further verification is needed to optimise the timing, dosing, and patient selection for ACEIs/ARBs in cancer radiotherapy.

## 5. Conclusions

ACEI use is associated with a reduction in radiation pneumonitis. Hypertensive patients with early-stage lung cancer can safely continue ACEIs during radiotherapy, but, due to insufficient evidence, the use of ACEIs/ARBs to prevent radiation pneumonitis in non-hypertensive patients remains unclear. Concurrent ACEI/ARB use during radiotherapy may reduce symptomatic brain radiation necrosis, alleviate prostate cancer symptoms, and improve functional independence in patients with glioblastoma. However, the survival outcomes vary in different types of cancer, with conflicting survival results for patients with lung cancer using ARBs. To confirm these findings and resolve inconsistencies, high-quality randomised trials are needed to provide more reliable evidence and improve clinical decision-making. Integrating ACEIs/ARBs into radiotherapy guidelines could improve patient outcomes and more effectively manage toxicity. Future research should explore the molecular mechanisms underlying the interactions between radiotherapy and ACEIs/ARBs and focus on optimising the ACEI/ARB dosing during radiotherapy to improve the clinical outcomes.

## Figures and Tables

**Table 1:** Inclusion and exclusion criteria of this study.

Component	Inclusion Criteria	Exclusion Criteria
Population and conditions	Patients aged 18 years and above. Patients diagnosed with histologically confirmed cancer (newly diagnosed or recurrent) and scheduled to receive radiotherapy.	Patients including paediatrics, children, adolescents, neonates, and infants. Studies including mixed age groups were excluded. Neoadjuvant radiotherapy or diagnostic radiology (e.g., X-rays, magnetic resonance images). Patients with cancer types not amenable to radiotherapy.
Intervention and comparator	Oral administration of ACEIs or ARBs, alone or in combination with other drugs, such as chemotherapy.	Non-concurrent use of ACEIs or ARBs and radiotherapy (i.e., not delivered during radiotherapy treatment). Single-dose intravenous therapy of ACEIs or ARBs.
Outcome	Survival outcomes, including overall survival, recurrence-free survival, recurrence rate, death rate, all-cause mortality, or remission. Radiotherapy-related side effects that occurred during or immediately after the radiotherapy.	Radiation-related toxicity that occurred before the administration of ACEIs or ARBs.
Study type	Human studies	Animal or in vitro studies.
Language	English	Other languages without English translation.
Publication	Full-text article on prospective or retrospective cohort study, cross-sectional study, or clinical trial.	Case–control study, case series, case report, systematic review, meta-analysis, conference abstract, abstract without full article, editorial, letter to editor, commentary, and grey literature.

(Note) ACEIs: angiotensin-converting enzyme inhibitors. ARBs: angiotensin receptor blockers. A mixed age group indicated that the population included paediatric patients.

**Table 2:** Characteristics of included studies.

Author, Year, Country	Cancer	Type of Radiation	Radiation Dose (Gy)	Number of Patients					Age of Patients (Year)	Ethnicity (Number of Patients)	Outcome Category
				Total	ACEI	ARB	ACEI /ARB ^§^	Non-User			
Wang, 2000, US [18]	Lung cancer	RT	Median (range): 65 (50, 80)	213	26			187	Median (range): 66 (37, 94)	Black (47)/white (165)/unknown (1)	RT-related side effects
Jenkins, 2011, UK [23]	NSCLC	Radical RT	Median (range): 54 (NA)	146	20	10		116	Median (range): 70 (47, 91)	NA	Survival outcomes
Kharofa, 2012, US [33]	Small cell and NSCLC	Thoracic irradiation	Median (range): 64 (NA)	162	62		10	100	Median (range): 65 (NA)	NA	Survival outcomes RT-related side effects
Wedlake, 2012, UK [38]	Pelvic malignancies	Radical pelvic RT	Median (range): ACEI users: 60 (45, 74); Non-users: 55.8 (20, 74)	237	39			198	Median (range): ACEI users: 72 (NA); Non-users: 68 (NA)	NA	Survival outcomes RT-related side effects
Wang, 2013, US [19]	NSCLC	Definitive RT	Median (range): ≥60 (NA)	413	65	49	111	302	Median (range): 66 (34, 88)	White (352)/nonwhite (61)	RT-related side effects
Harder, 2015, US [20]	Primary lung cancer	SBRT	Median (range): 54 (NA)	257	70		35	187	Mean: 74.7 ± 0.6 *	NA	Survival outcomes RT-related side effects
Januel, 2015, France [37]	Supratentorial glioblastoma	RT	Range: 56, 60	81			26	55	Median ± SD: ACEI/ARB users: 65 ± 10; Non-users: 63 ± 9	NA	Survival outcomes RT-related side effects
Alashkham, 2016, UK [16]	Localised or locally advanced adenocarcinoma of the prostate	Radical RT	Median (range): 54 (45, 57)	308	102			206	Mean ± SD: 68.91 ± 5.67	NA	RT-related side effects
Bracci, 2016, Italy [14]	NSCLC	SBRT	Range: 23, 45	158	33	28	61	97	Median (range): 72 (25, 90)	NA	RT-related side effects
Alite, 2018, US [21]	Lung cancer or oligometastases	SBRT	Range: 48, 60	189	49		22	140	Median (range): 71 (29, 90)	NA	RT-related side effects
Chowdhary, 2018, US [36]	Brain metastases	Stereotactic radiosurgery	Median (range): 18 (15, 21)	111			32	79	NA	NA	Survival outcomes RT-related side effects
Small, 2018, US [35]	Small cell and NSCLC	RT	Median (range): ≥45 (NA)	20	7			13	Median (range): ACEI users: 64 (46, 75); Non-users: 67 (42, 87)	NA	RT-related side effects
Sio, 2019, US [34]	Advanced NSCLC	Curative thoracic RT	Median (range): ≥45 (NA)	21	11			10	Median (range): ACEI users: 62 (49, 87); Non-users: 62 (54, 83)	White (20)/nonwhite (1)	RT-related side effects
Kerns, 2022, UK [17]	Prostate cancer	Potentially curative RT	Range: 40, 75	1693	438			1255	Median (range): 70 (42, 86)	White (1622)/Black or African American (42)/Other or not specified (29)	RT-related side effects
Maloney, 2022, US [26]	Early-stage lung cancer	SBRT	Median (IQR): 115.5 (100.0, 132.0)	247		24		223	Mean ± SD: 73.5 ± 8.4	NA	Survival outcomes RT-related side effects
Zheng, 2023, China [15]	Lung cancer	Thoracic radiation	Median (range): 60 (NA)	320	8	54	62	258	Mean ± SD: 64.9 ± 9.6	Chinese	RT-related side effects

(Note) US: United States. UK: United Kingdom. NSCLC: non-small-cell lung cancer. RT: radiotherapy. SBRT: stereotactic body radiation therapy. NA: not available. IQR: interquartile range. ACEI: angiotensin-converting enzyme inhibitor. ARB: angiotensin receptor blocker. ^§^ ACEI/ARB represents patients using ACEIs and/or ARBs across various studies. * standard error of the mean. SD: standard deviation. Various adverse events were reported, including radiation pneumonitis (n = 9) [14,15,18,19,20,21,26,33,35], brain radiation necrosis (n = 1) [36], functional independence (n = 1) [37], pulmonary fibrosis (n = 1) [26], proctitis (n = 1) [16], kidney injury (n = 1) [34], haematuria and rectal bleeding (n = 1) [17], and hypotension (n = 1) [34]. Survival outcomes were reported as overall survival time (n = 6) [20,23,26,33,36,37], recurrence-free survival time (n = 2) [26,37], overall survival rate at two years (n = 1) [20], total recurrence and total death rate (n = 1) [26], and death due to disease progression and cancer-related treatment toxicity rate (n = 1) [38].

**Table 3:** Odds ratio for ≥grade 2 radiation pneumonitis in ACEI/ARB users compared to non-users.

Study	Type of Cancer	Follow-Up (Months)	Event Rate	Odds Ratio (95%CI)	Effect Size
≥Grade 2 radiation pneumonitis					
ACEIs vs. non-user					
Wang (2000) [18]	Lung cancer	Median (range): 13.9 (1–54)	4/26 vs. 22/187	1.36 (0.43, 4.33)	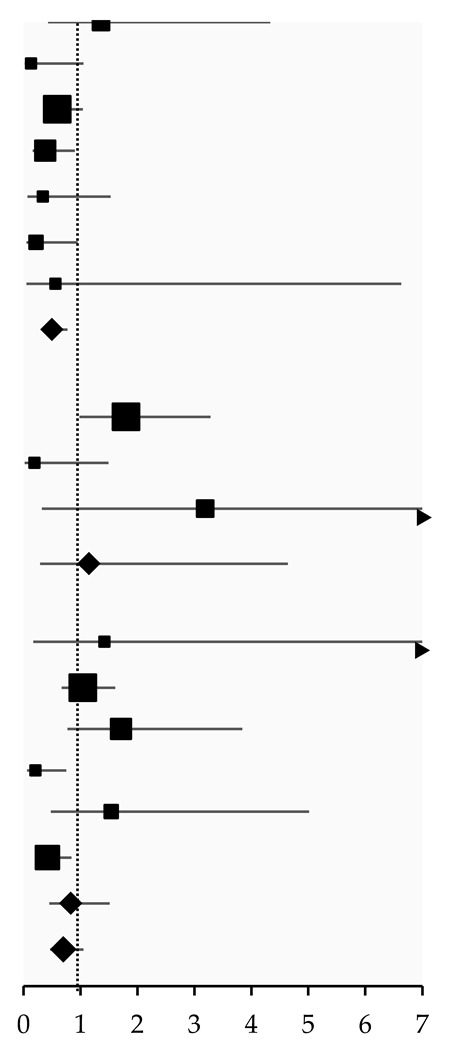
Kharofa (2012) [33]	Small cell and NSCLC	NA	1/62 vs. 11/100	0.13 (0.02, 1.05)	
Wang (2013) [19]	NSCLC	Median (range): 18 (NA)	22/65 vs. 161/348	0.59 (0.34, 1.04)	
Harder (2015) [20]	Primary lung cancer	Median (range): 25.2 (NA)	7/70 vs. 42/187	0.38 (0.16, 0.90) *	
Bracci (2016) [14]	NSCLC	Median (range): 13.8 (3.2–55.0)	2/33 vs. 20/125	0.34 (0.07, 1.53)	
Alite (2018) [21]	Lung cancer or oligometastases	Median (range): 24.8 (NA)	2/49 vs. 23/140	0.22 (0.05, 0.95) *	
Small (2018) [35]	Small cell and NSCLC	Median (range): 16.5 (3.4–30.0)	1/7 vs. 3/13	0.56 (0.05, 6.63)	
Subgroup			39/312 vs. 282/1100	0.50 (0.32, 0.77) *, *I*^2^ = 10%	
ARBs vs. non-user					
Wang (2013) [19]	NSCLC	Median (range): 18 (NA)	28/49 vs. 155/364	1.80 (0.98, 3.28)	
Bracci (2016) [14]	NSCLC	Median (range): 13.8 (3.2–55.0)	1/28 vs. 21/130	0.19 (0.02, 1.49)	
Maloney (2022) [26]	Early-stage lung cancer	NA	1/24 vs. 3/223	3.19 (0.32, 31.92)	
Subgroup			30/101 vs. 179/717	1.15 (0.29, 4.64), *I*^2^ = 56%	
ACEIs/ARBs vs. non-user					
Kharofa (2012) [33]	Small cell and NSCLC	NA	1/10 vs. 11/152	1.42 (0.17, 12.29)	
Wang (2013) [19]	NSCLC	Median (range): 18 (NA)	50/111 vs. 133/302	1.04 (0.67, 1.61)	
Harder (2015) [20]	Primary lung cancer	At 12 months	10/35 vs. 42/222	1.71 (0.77, 3.84)	
Bracci (2016) [14]	NSCLC	Median (range): 13.8 (3.2–55.0)	3/61 vs. 19/97	0.21 (0.06, 0.75) *	
Alite (2018) [21]	Lung cancer or oligometastases	Median (range): 24.8 (NA)	4/22 vs. 21/167	1.54 (0.48, 5.01)	
Zheng (2023) [15]	Lung cancer	Median (range): 12.5 (0.5–56.7)	11/62 vs. 88/258	0.42 (0.21, 0.84) *	
Subgroup			79/301 vs. 314/1198	0.83 (0.45, 1.51), *I*^2^ = 63%	
Overall			148/714 vs. 775/3015	0.70 (0.47, 1.05), *I*^2^ = 58%	

(Note) ACEIs: angiotensin-converting enzyme inhibitors. ARBs: angiotensin receptor blockers. NSCLC: non-small-cell lung cancer. NA: not available. CI: confidence interval. * significant difference. The x-axis values of the effect size represent the odds ratio and 95% confidence interval of each study and the overall synthesised results.

**Table 4:** Odds ratio for freedom from symptomatic (≥grade 2) radiation pneumonitis in ACEI/ARB users compared to non-users.

Study	Cancer	Follow-Up	Event Rate	Odds Ratio (95%CI), *I*^2^	Effect Size
ACEIs vs. non-user					
Kharofa (2012) [33]	Small cell and NSCLC	At 6 months	61/62 vs. 89/100	7.54 (0.95, 59.92)	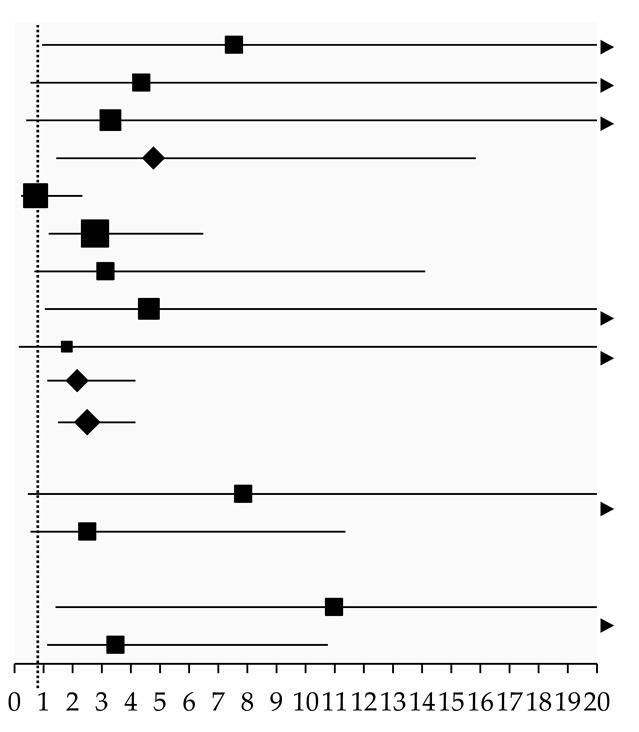
Bracci (2016) [14]	NSCLC	At 6 months	32/33 vs. 110/125	4.36 (0.55, 34.31)	
Alite (2018) [21]	Lung cancer or oligometastases	At 6 months	48/49 vs. 131/140	3.30 (0.41, 26.72)	
Subgroup		At 6 months	141/144 vs. 330/365	4.78 (1.44, 15.84) *, *I*^2^ < 0.1%	
Wang (2000) [18]	Lung cancer	At 12 months	22/26 vs. 165/187	0.73 (0.23, 2.33)	
Harder (2015) [20]	Primary lung cancer	At 12 months	63/70 vs. 143/187	2.77 (1.18, 6.48)	
Bracci (2016) [14]	NSCLC	At 12 months	31/33 vs. 104/125	3.13 (0.69, 14.09)	
Alite (2018) [21]	Lung cancer or oligometastases	At 12 months	47/49 vs. 117/140	4.62 (1.05, 20.38)	
Small (2018) [35]	Small cell and NSCLC	At 12 months	6/7 vs. 10/13	1.80 (0.15, 21.48)	
Subgroup		At 12 months	169/185 vs. 539/652	2.16 (1.13, 4.15) *, *I*^2^ = 19%	
Overall			310/329 vs. 869/1017	2.50 (1.50, 4.15) *, *I*^2^ < 0.1%	
ARBs vs. non-user					
Bracci (2016) [14]	NSCLC	At 6 months	28/28 vs. 114/130	7.86 (0.46, 135.20)	
Bracci (2016) [14]	NSCLC	At 12 months	26/28 vs. 109/130	2.50 (0.55, 11.36)	
ACEIs/ARBs vs. non-user					
Bracci (2016) [14]	NSCLC	At 6 months	60/61 vs. 82/97	10.98 (1.41, 85.38) *	
Bracci (2016) [14]	NSCLC	At 12 months	57/61 vs. 78/97	3.47 (1.12, 10.76) *	

(Note) ACEIs: angiotensin-converting enzyme inhibitors. ARBs: angiotensin receptor blockers. NSCLC: non-small-cell lung cancer. CI: confidence interval. * significant difference. The x-axis values of the effect size represent the odds ratio and 95% confidence interval of each study and the overall synthesised results.

**Table 5:** Odds ratios for brain radiation necrosis, functional independence, proctitis, kidney injury, haematuria, rectal bleeding, pulmonary fibrosis, and hypotension in ACEI/ARB users compared to non-users.

Study	Cancer	Comparison	Follow-Up (Months)	Event Rate	Odds Ratio (95%CI)
Symptomatic radiation necrosis					
Chowdhary (2018) [36]	Brain metastases	ACEIs/ARBs vs. non-users	Median (range): ACEI/ARB users: 8.7 (NA); Non-users:13.9 (NA)	2/32 vs. 27/79	0.13 (0.03, 0.58) *
Radiation necrosis rate					
Chowdhary (2018) [36]	Brain metastases	ACEIs/ARBs vs. non-users	At 1 year	4/32 vs. 17/79	0.52 (0.16, 1.69)
Symptomatic radiation necrosis rate					
Chowdhary (2018) [36]	Brain metastases	ACEIs/ARBs vs. non-users	At 1 year	1/32 vs. 20/79	0.10 (0.01, 0.74) *
Functionally independent rate					
Januel (2015) [37]	Supratentorial glioblastoma	ACEIs/ARBs vs. non-users	At 1 month	26/26 vs. 43/55	14.51 (0.82, 256.10)
Januel (2015) [37]	Supratentorial glioblastoma	ACEIs/ARBs vs. non-users	At 6 months	22/26 vs. 31/55	4.26 (1.29, 14.01) *
Proctitis					
Alashkham (2016) [16]	Localised or locally advanced adenocarcinoma of the prostate	ACEIs vs. non-users	Mean ± SD: 40.56 ± 23.4	34/102 vs. 148/206	0.20 (0.12, 0.33) *
Grade 2 acute kidney injury					
Sio (2019) [34]	Advanced NSCLC	ACEIs vs. non-users	NA	1/11 vs. 0/10	2 (0.06, 66.64)
Developing haematuria					
Kerns (2022) [17]	Prostate cancer	ACEIs vs. non-users	At 4 years	21/438 vs. 207/1255	0.25 (0.16, 0.41) *
Haematuria					
Kerns (2022) [17]	Prostate cancer	ACEIs vs. non-users	Median (range): 24 (NA)	3/33 vs. 220/1222	0.46 (0.14, 1.51)
Rectal bleeding					
Kerns (2022) [17]	Prostate cancer	ACEIs vs. non-users	Median (range): 24 (NA)	75/438 vs. 435/1255	0.39 (0.30, 0.51) *
Pulmonary fibrosis rate					
Maloney (2022) [26]	Early-stage lung cancer	ARBs vs. non-users	NA	4/24 vs. 36/223	1.04 (0.34, 3.22)
Grade 2 hypotension					
Sio (2019) [34]	Advanced NSCLC	ACEIs vs. non-users	NA	4/11 vs. 2/10	2.3 (0.32, 16.51)

(Note) NSCLC: non-small-cell lung cancer. ACEIs: angiotensin-converting enzyme inhibitors. ARBs: angiotensin receptor blockers. NA: not available. SD: standard deviation. CI: confidence interval. * significant difference.

## Data Availability

Research data are available upon request from the lead author.

## Supplementary Materials

The following supporting information can be downloaded at: https://www.mdpi.com/article/10.3390/ph18010105/s1, Table S1. PRISMA 2020 Checklist, File S1. Electronic Search Strategy, Table S2. The Risk of Bias Assessment for the Two Randomised Controlled Trials, Table S3. The Risk of Bias Assessment for the 14 Cohort Studies, Table S4. Survival Outcome.
