# Supplementary material for: The Effect of the Concurrent Use of Angiotensin-Converting Enzyme Inhibitors or Receptor Blockers on Toxicity and Outcomes in Patients Treated with Radiotherapy: A Systematic Review and Meta-Analysis
Source: Pharmaceuticals (Basel). 2025 Jan 16;18(1):105. doi: 10.3390/ph18010105 (PMC11769270; doi:10.3390/ph18010105)
Supplement: Supplementary file 1 [file pharmaceuticals-18-00105-s001.zip › pharmaceuticals-3409522-supplementary.pdf]

Table S1. PRISMA 2020 Checklist

Section and Topic	Item #	Checklist Item	Location Where Item Is Reported
TITLE			
Title	1	Identify the report as a systematic review.	1
ABSTRACT			
Abstract	2	See the PRISMA 2020 for Abstracts checklist.	1
INTRODUCTION			
Rationale	3	Describe the rationale for the review in the context of existing knowledge.	2
Objectives	4	Provide an explicit statement of the objective(s) or question(s) the review addresses.	2
METHODS			
Eligibility criteria	5	Specify the inclusion and exclusion criteria for the review and how studies were grouped for the syntheses.	2–5, Table 1
Information sources	6	Specify all databases, registers, websites, organizations, reference lists and other sources searched or consulted to identify studies. Specify the date when each source was last searched or consulted.	2–5
Search strategy	7	Present the full search strategies for all databases, registers and websites, including any filters and limits used.	2–5, File S1
Selection process	8	Specify the methods used to decide whether a study met the inclusion criteria of the review, including how many reviewers screened each record and each report retrieved, whether they worked independently, and, if applicable, details of automation tools used in the process.	2–5
Data collection process	9	Specify the methods used to collect data from reports, including how many reviewers collected data from each report, whether they worked independently, any processes for obtaining or confirming data from study investigators, and, if applicable, details of automation tools used in the process.	2–5
Data items	10a	List and define all outcomes for which data were sought. Specify whether all results that were compatible with each outcome domain in each study were sought (e.g., for all measures, time points, analyses), and, if not, the methods used to decide which results to collect.	2–5
	10b	List and define all other variables for which data were sought (e.g., participant and intervention characteristics, funding sources). Describe any assumptions made about any missing or unclear information.	2–5
Study risk of bias assessment	11	Specify the methods used to assess the risk of bias in the included studies, including details of the tool(s) used, how many reviewers assessed each study and whether they worked independently, and, if applicable, details of automation tools used in the process.	2–5
Effect measures	12	Specify for each outcome the effect measure(s) (e.g., risk ratio, mean difference) used in the synthesis or presentation of results.	2–5
Synthesis methods	13a	Describe the processes used to decide which studies were eligible for each synthesis (e.g., tabulating the study intervention characteristics and comparing against the planned groups for each synthesis (item #5)).	2–5

	13b	Describe any methods required to prepare the data for presentation or synthesis, such as handling of missing summary statistics or data conversions.	2–5
	13c	Describe any methods used to tabulate or visually display results of individual studies and syntheses.	2–5
	13d	Describe any methods used to synthesize results and provide a rationale for the choice(s). If a meta-analysis was performed, describe the model(s), method(s) to identify the presence and extent of statistical heterogeneity, and software package(s) used.	2–5
	13e	Describe any methods used to explore possible causes of heterogeneity among study results (e.g., subgroup analysis, meta-regression).	2–5
	13f	Describe any sensitivity analyses conducted to assess the robustness of the synthesized results.	2–5
Reporting bias assessment	14	Describe any methods used to assess the risk of bias due to missing results in a synthesis (arising from reporting biases).	2–5
Certainty assessment	15	Describe any methods used to assess certainty (or confidence) in the body of evidence for an outcome.	2–5
RESULTS			
Study selection	16a	Describe the results of the search and selection process, from the number of records identified in the search to the number of studies included in the review, ideally using a flow diagram.	5–6, Figure 1
	16b	Cite studies that might appear to meet the inclusion criteria but which were excluded, and explain why they were excluded.	Figure 1
Study characteristics	17	Cite each included study and present its characteristics.	5–9, Table 2
Risk of bias in studies	18	Present assessments of risk of bias for each included study.	10, Tables S2 and S3
Results of individual studies	19	For all outcomes, present, for each study: (a) summary statistics for each group (where appropriate) and (b) an effect estimate and its precision (e.g., confidence/credible interval), ideally using structured tables or plots.	5–13, Tables 3–5, Tables S4
Results of syntheses	20a	For each synthesis, briefly summarize the characteristics and risk of bias among contributing studies.	10, Tables S2 and S3
	20b	Present results of all statistical syntheses conducted. If a meta-analysis was done, present for each the summary estimate and its precision (e.g., confidence/credible interval) and measures of statistical heterogeneity. If comparing groups, describe the direction of the effect.	5–13, Tables 3–5, Tables S4
	20c	Present results of all investigations of possible causes of heterogeneity among study results.	5–13
	20d	Present results of all sensitivity analyses conducted to assess the robustness of the synthesized results.	5–13
Reporting biases	21	Present assessments of risk of bias due to missing results (arising from reporting biases) for each synthesis assessed.	5–13
Certainty of evidence	22	Present assessments of certainty (or confidence) in the body of evidence for each outcome assessed.	5–13
DISCUSSION			
Discussion	23a	Provide a general interpretation of the results in the context of other evidence.	14

	23b	Discuss any limitations of the evidence included in the review.	14–16
	23c	Discuss any limitations of the review processes used.	14–16
	23d	Discuss implications of the results for practice, policy, and future research.	14–16
OTHER INFORMATION			
Registration and protocol	24a	Provide registration information for the review, including register name and registration number, or state that the review was not registered.	2–5
	24b	Indicate where the review protocol can be accessed or state that a protocol was not prepared.	2–5
	24c	Describe and explain any amendments to information provided at registration or in the protocol.	2–5
Support	25	Describe sources of financial or non-financial support for the review and the role of the funders or sponsors in the review.	16
Competing interests	26	Declare any competing interests of review authors.	17
Availability of data, code and other materials	27	Report which of the following are publicly available and where they can be found: template data collection forms; data extracted from included studies; data used for all analyses; analytic code; any other materials used in the review.	17

File S1. Electronic Search Strategy

MEDLINE

1. exp Radiotherapy/
2. exp Radiation/
3. exp Chemoradiotherapy/
4. exp Radiotherapy, Computer-Assisted/
5. Stereotactic body radiation therapy.mp.
6. Stereotactic radiosurgery.mp. or Radiosurgery/
7. Definitive Radiation Therapy.mp.
8. Thoracic Irradiation.mp.
9. Total body irradiation.mp. or Whole-Body Irradiation/
10. Radiotherap*.mp.
11. Radiother*.mp.
12. Radiat*.mp.
13. Irradiat*.mp.
14. (Radiochemo* or Chemoradio*).mp. [mp = title, book title, abstract, original title, name of substance word, subject heading word, floating sub-heading word, keyword heading word, organism supplementary concept word, protocol supplementary concept word, rare disease supplementary concept word, unique identifier, synonyms, population supplementary concept word, anatomy supplementary concept word]
15. Radiation therap*.mp.
16. 1 or 2 or 3 or 4 or 5 or 6 or 7 or 8 or 9 or 10 or 11 or 12 or 13 or 14 or 15
17. exp Angiotensin-Converting Enzyme Inhibitors/
18. ((angiotensin$ or dipeptidyl$ or kininase ii) adj3 (convert$ or enzyme or inhibit$ or recept$ or block$)).tw,kf.
19. Angiotensin converting enzyme inhibit$.tw.
20. (ace adj2 inhibit$).tw,kf.
21. (ACE or ACE1 or ACEI or ACE-I or ACEs).tw,kf.
22. Benazepril.mp.
23. Lotensin.mp.
24. Captopril/
25. Capoten.mp.
26. Enalapril/
27. Vasotec.mp.
28. Fosinopril/
29. Monopril.mp.
30. Lisinopril/
31. Prinivil.mp.
32. Zestril.mp.
33. Moexipril.mp.
34. Univasc.mp.
35. Perindopril/
36. Coversyl.mp.
37. Quinapril/
38. Accupril.mp.
39. Ramipril/
40. Triapin.mp.
41. Triapin Mite.mp.
42. Tritace.mp.

43. Trandolapril.mp.
44. Mavik.mp.
45. Cilazapril/
46. Spirapril.mp.
47. Delapril.mp.
48. Zofenopril.mp.
49. Imidapril.mp.
50. Alacepril.mp.
51. Altiopril.mp.
52. Ancovenin.mp.
53. Ceranapril.mp.
54. Ceronapril.mp.
55. Deacetylalacepril.mp.
56. Derapril.mp.
57. Enalaprilat/
58. Enalapril$.mp.
59. Epicaptopril.mp.
60. Fasidotril.mp.
61. Fasidotril$.mp.
62. Foroxymithine.mp.
63. Fosinopril$.mp.
64. Gemopatrilat.mp.
65. Imidapril.mp.
66. Imidapril$.mp.
67. Indolapril.mp.
68. Libenzapril.mp.
69. Moexipril$.mp.
70. Moveltipril.mp.
71. Omapatrilat.mp.
72. Pentopril$.mp.
73. Perindopril$.mp.
74. Pivopril.mp.
75. Quinapril$.mp.
76. Ramipril$.mp.
77. Rentiapril.mp.
78. Saralasin/
79. S-nitrosocaptopril.mp.
80. Spirapril$.mp.
81. Temocapril$.mp.
82. Teprotide/
83. Trandolapril$.mp.
84. Utibapril$.mp.
85. Zabicipril$.mp.
86. Zofenopril$.mp.
87. Altace.mp.
88. Univas.mp.
89. exp Angiotensin II Type 1 Receptor Blockers/
90. exp Angiotensin Receptor Antagonists/
91. Receptors, Angiotensin/
92. Angiotensin II receptor blocker$.tw.
93. Angiotensin 2 receptor blocker$.tw.

94. Angiotensin II receptor antagonist$.tw.
95. Angiotensin 2 receptor antagonists$.tw.
96. AT 2 receptor block$.tw.
97. AT 2 receptor antagon$.tw.
98. Angiotensin receptor antagonist$.tw.
99. (angiotensin adj3 receptor antagon$).tw,kf.
100. (angiotensin adj3 receptor block$).tw,kf.
101. (ARB or ARBs).tw,kf.
102. Azilsartan.mp.
103. Edarbi.mp.
104. Candesartan.mp.
105. Blopress.mp.
106. Atacand.mp.
107. Amias.mp.
108. Ratacand.mp.
109. Eprosartan.mp.
110. Teveten.mp.
111. Irbesartan/
112. Avapro.mp.
113. Losartan/
114. Cozaar.mp.
115. Olmesartan.mp.
116. Benicar.mp.
117. Telmisartan/
118. Micardis.mp.
119. Valsartan/
120. Diovan.mp.
121. Entresto.mp.
122. Exforge.mp.
123. Byvalson.mp.
124. Embusartan.mp.
125. Forasartan.mp.
126. KT3-671.mp.
127. Milfasartan.mp.
128. Saprisartan.mp.
129. Tasosartan.mp.
130. Zolasartan.mp.
131. 17 or 18 or 19 or 20 or 21 or 22 or 23 or 24 or 25 or 26 or 27 or 28 or 29 or 30 or 31 or 32 or 33 or 34 or 35 or 36 or 37 or 38 or 39 or 40 or 41 or 42 or 43 or 44 or 45 or 46 or 47 or 48 or 49 or 50 or 51 or 52 or 53 or 54 or 55 or 56 or 57 or 58 or 59 or 60 or 61 or 62 or 63 or 64 or 65 or 66 or 67 or 68 or 69 or 70 or 71 or 72 or 73 or 74 or 75 or 76 or 77 or 78 or 79 or 80 or 81 or 82 or 83 or 84 or 85 or 86 or 87 or 88 or 89 or 90 or 91 or 92 or 93 or 94 or 95 or 96 or 97 or 98 or 99 or 100 or 101 or 102 or 103 or 104 or 105 or 106 or 107 or 108 or 109 or 110 or 111 or 112 or 113 or 114 or 115 or 116 or 117 or 118 or 119 or 120 or 121 or 122 or 123 or 124 or 125 or 126 or 127 or 128 or 129 or 130
132. 16 and 131
133. limit 132 to (english language and humans)

EMBASE

1. exp Radiotherapy/
2. exp Radiation/

3. exp Chemoradiotherapy/
4. exp Radiotherapy, Computer-Assisted/
5. Stereotactic body radiation therapy.mp.
6. Stereotactic radiosurgery.mp. or Radiosurgery/
7. Definitive Radiation Therapy.mp.
8. Thoracic Irradiation.mp.
9. Total body irradiation.mp. or Whole-Body Irradiation/
10. Radiotherap*.mp.
11. Radiother*.mp.
12. Radiat*.mp.
13. Irradiat*.mp.
14. (Radiochemo* or Chemoradio*).mp. [mp = title, abstract, heading word, drug trade name, original title, device manufacturer, drug manufacturer, device trade name, keyword heading word, floating subheading word, candidate term word]
15. Radiation therap*.mp.
16. 1 or 2 or 3 or 4 or 5 or 6 or 7 or 8 or 9 or 10 or 11 or 12 or 13 or 14 or 15
17. exp Angiotensin-Converting Enzyme Inhibitors/
18. ((angiotensin$ or dipeptidyl$ or kininase ii) adj3 (convert$ or enzyme or inhibit$ or recept$ or block$)).tw,kf.
19. Angiotensin converting enzyme inhibit$.tw.
20. (ace adj2 inhibit$).tw,kf.
21. (ACE or ACE1 or ACEI or ACE-I or ACEs).tw,kf.
22. Benazepril.mp.
23. Lotensin.mp.
24. Captopril/
25. Capoten.mp.
26. Enalapril/
27. Vasotec.mp.
28. Fosinopril/
29. Monopril.mp.
30. Lisinopril/
31. Prinivil.mp.
32. Zestril.mp.
33. Moexipril.mp.
34. Univasc.mp.
35. Perindopril/
36. Coversyl.mp.
37. Quinapril/
38. Accupril.mp.
39. Ramipril/
40. Triapin.mp.
41. Triapin Mite.mp.
42. Tritace.mp.
43. Trandolapril.mp.
44. Mavik.mp.
45. Cilazapril/
46. Spirapril.mp.
47. Delapril.mp.
48. Zofenopril.mp.
49. Imidapril.mp.
50. Alacepril.mp.

51. Altiopril.mp.
52. Ancovenin.mp.
53. Ceranapril.mp.
54. Ceronapril.mp.
55. Deacetylalacepril.mp.
56. Derapril.mp.
57. Enalaprilat/
58. Enalapril$.mp.
59. Epicaptopril.mp.
60. Fasidotril.mp.
61. Fasidotril$.mp.
62. Foroxymithine.mp.
63. Fosinopril$.mp.
64. Gemopatrilat.mp.
65. Imidapril.mp.
66. Imidapril$.mp.
67. Indolapril.mp.
68. Libenzapril.mp.
69. Moexipril$.mp.
70. Moveltipril.mp.
71. Omapatrilat.mp.
72. Pentopril$.mp.
73. Perindopril$.mp.
74. Pivopril.mp.
75. Quinapril$.mp.
76. Ramipril$.mp.
77. Rentiapril.mp.
78. Saralasin/
79. S-nitrosocaptopril.mp.
80. Spirapril$.mp.
81. Temocapril$.mp.
82. Teprotide/
83. Trandolapril$.mp.
84. Utibapril$.mp.
85. Zabicipril$.mp.
86. Zofenopril$.mp.
87. Altace.mp.
88. Univas.mp.
89. exp Angiotensin II Type 1 Receptor Blockers/
90. exp Angiotensin Receptor Antagonists/
91. Receptors, Angiotensin/
92. Angiotensin II receptor blocker$.tw.
93. Angiotensin 2 receptor blocker$.tw.
94. Angiotensin II receptor antagonist$.tw.
95. Angiotensin 2 receptor antagonists$.tw.
96. AT 2 receptor block$.tw.
97. AT 2 receptor antagon$.tw.
98. Angiotensin receptor antagonist$.tw.
99. (angiotensin adj3 receptor antagon$).tw,kf.
100. (angiotensin adj3 receptor block$).tw,kf.
101. (ARB or ARBs).tw,kf.

102. Azilsartan.mp.
103. Edarbi.mp.
104. Candesartan.mp.
105. Blopress.mp.
106. Atacand.mp.
107. Amias.mp.
108. Ratacand.mp.
109. Eprosartan.mp.
110. Teveten.mp.
111. Irbesartan/
112. Avapro.mp.
113. Losartan/
114. Cozaar.mp.
115. Olmesartan.mp.
116. Benicar.mp.
117. Telmisartan/
118. Micardis.mp.
119. Valsartan/
120. Diovan.mp.
121. Entresto.mp.
122. Exforge.mp.
123. Byvalson.mp.
124. Embusartan.mp.
125. Forasartan.mp.
126. KT3-671.mp.
127. Milfasartan.mp.
128. Saprisartan.mp.
129. Tasosartan.mp.
130. Zolasartan.mp.
131. 17 or 18 or 19 or 20 or 21 or 22 or 23 or 24 or 25 or 26 or 27 or 28 or 29 or 30 or 31 or 32 or 33 or 34 or 35 or 36 or 37 or 38 or 39 or 40 or 41 or 42 or 43 or 44 or 45 or 46 or 47 or 48 or 49 or 50 or 51 or 52 or 53 or 54 or 55 or 56 or 57 or 58 or 59 or 60 or 61 or 62 or 63 or 64 or 65 or 66 or 67 or 68 or 69 or 70 or 71 or 72 or 73 or 74 or 75 or 76 or 77 or 78 or 79 or 80 or 81 or 82 or 83 or 84 or 85 or 86 or 87 or 88 or 89 or 90 or 91 or 92 or 93 or 94 or 95 or 96 or 97 or 98 or 99 or 100 or 101 or 102 or 103 or 104 or 105 or 106 or 107 or 108 or 109 or 110 or 111 or 112 or 113 or 114 or 115 or 116 or 117 or 118 or 119 or 120 or 121 or 122 or 123 or 124 or 125 or 126 or 127 or 128 or 129 or 130
132. 16 and 131
133. limit 132 to (human and english)

Web of Science

(Radiotherapy or Radiation or Chemoradiotherapy or Stereotactic body radiation therapy or Stereotactic radiosurgery or Radiosurgery or Definitive Radiation Therapy or Thoracic Irradiation or Total Body Irradiation or Whole-Body Irradiation or Radiotherapy-Computer-Assisted or Radiotherap* or Radiother* or Radiat* or Irradiat* or Radiochemo* or Chemoradio* or Radiation therap*) and (Angiotensin-Converting Enzyme Inhibitors or Angiotensin Converting Enzyme Inhibit* or ACE or ACE1 or ACEI or ACE-I or ACEs or Benazepril or lotensis or Captopril or caroten or Enalapril or vasotech or Fosinopril or monocril or Lisinopril or prinvil or zentral or Moexipril or univasf or Perindopril or coversly or Quinapril or accuprep or Ramipril or lopate or Ramipril or triazin or triazin Mite or tritane or Trandolapril or malik or cilazapril or spirapril or

delapril or zofenopril or imidapril or alacepril or alatriopril or antivenin or ceranapril or ceranapril or desacetylalacepril or delapril or enalaprilat or enalapril* or epicaptopril or fasidopril or fasidopril* or foroximithine or fosinopril* or gemopatrilat or idrapril or imidapril or imidapril* or indalapril or libenzapril or moexipril* or moveltipril or omapatrilat or pentopril* or perindopril* or pivoxil or quinapril* or ramipril* or renipril or saralasin or s-nitrosocaptopril or spirapril* or temocapril* or teproside or trandolapril$ or utibapril$ or zabicipril* or zofenopril* or acton or alsace or unidas or Angiotensin II Type 1 Receptor Blockers or Angiotensin Receptor Antagonists or angiotensin II receptor blocker* or angiotensin 2 receptor blocker* or angiotensin II receptor antagonist* or angiotensin 2 receptor antagonists* or AT 2 receptor block* or AT 2 receptor antagon* or angiotensin receptor antagonist* or ARB or ARBs or Azilsartan or edapbi or Candesartan or biopress or acacand or alias or radicand or Eprosartan or tevenen or Irbesartan or avipro or Losartan or cozar or Olmesartan or beninar or Telmisartan or micardia or Valsartan or dioxan or entrepot or enforce or byalison or abitesartan or elmisartan or embusartan or fimasartan or KT3-671 or milfasartan or saprisartan or tasosartan or zolarsartan)

Scopus

(radiotherapy OR radiation OR chemoradiotherapy OR radiosurgery OR irradiation OR radiotherapy-computer-assisted OR radiotherap* OR radiother* OR radiat* OR irradiat* OR radiochemo* OR chemoradio*) AND (angiotensin AND converting AND enzyme AND inhibitors) OR (angiotensin AND converting AND ad AND enzyme) OR ace OR acei OR arb

Table S2. The Risk of Bias Assessment for the Two Randomised Controlled Trials

Author, Year	Bias Arising from the Randomisation Process	Bias Due to Deviations from Intended Intervention	Bias Due to Missing Outcome Data	Bias in the Measurement of the Outcome	Bias in the Selection of the Reported Result	Overall
Small, 2018 [35]	Low	Low	Some concerns	Low	Low	Some concerns
Sio, 2019 [34]	Low	Low	Low	Low	Some concerns	Some concerns

(Note) The Risk of Bias Tool for Randomised Trials (RoB 2) was applied. Background colours: green—low risk of bias; yellow—some concerns; and red—high risk of bias.

Table S3. The Risk of Bias Assessment for the 14 Cohort Studies

Author, Year	Bias Due to Confounding	Bias Due to the Selection of Participants	Bias in the Classification of Interventions	Bias Due to Deviations from Intended Interventions	Bias Due to Missing Data	Bias in the Measurement of Outcomes	Bias in the Selection of the Reported Result	Overall
Wang, 2000 [18]	Moderate	Low	Low	Low	Low	Low	Low	Moderate
Jenkins, 2011 [23]	Moderate	Low	Low	Low	Low	Low	Serious	Serious
Kharofa, 2012 [33]	Moderate	Serious	Low	Low	Low	Low	Moderate	Serious

Wedlake, 2012 [38]	Moderate	Low	Low	Low	Moderate	Low	Moderate	Moderate
Wang, 2013 [19]	Moderate	Low	Low	Low	Low	Low	Low	Moderate
Harder, 2015 [20]	Moderate	Low	Low	Low	Low	Low	Low	Moderate
Januel, 2015 [37]	Moderate	Low	Low	Low	Low	Low	Moderate	Moderate
Alashkham, 2016 [16]	Moderate	Low	Low	Low	Low	Low	Low	Moderate
Bracci, 2016 [14]	Low	Low	Low	Low	Low	Low	Low	Low
Alite, 2018 [21]	Low	Low	Low	Low	Low	Low	Low	Low
Chowdhary, 2018 [36]	Moderate	Low	Low	Low	Low	Low	Low	Moderate
Kerns, 2022 [17]	Moderate	Low	Low	Low	Low	Low	Low	Moderate
Maloney, 2022 [26]	Moderate	Low	Low	Low	Low	Low	Low	Moderate
Zheng, 2023 [15]	Moderate	Low	Low	Low	Low	Low	Low	Moderate

(Note) The Risk of Bias in Non-Randomised Studies of Interventions (ROBINS-I) tool was applied. Background colours: green—low risk of bias (the study is comparable to a well-performed randomised trial concerning this domain); yellow—moderate risk of bias (the study is sound for a non-randomised study concerning this domain but cannot be considered comparable to a well-performed randomised trial); orange—serious risk of bias (the study has some important problems); and red—critical risk of bias (the study is too problematic to provide any useful evidence on the effects of intervention).

Table S4. Survival Outcome

Study	Cancer	Comparison	No. of Patients	Median Survival Time (Months)	Median Time Difference Between Exposed and Non-Exposed Groups (Months)
**Overall survival time**					
Jenkins (2011)	Non-small-cell lung cancer	ACEIs vs. non-users	20 vs. 116	18 vs. 15	3
Kharofa (2012)	Small cell and non-small cell lung cancer	ACEIs vs. non-users	62 vs. 100	19 vs. 22	−3
Harder (2015)	Primary lung cancer	ACEIs vs. non-users	70 vs. 187	29.3 vs. 25.9	3.4
Januel (2015)	Supratentorial glioblastoma	ACEIs/ARBs vs. non-users	26 vs. 55	16.9 vs. 13.1	3.8
Chowdhary (2018)	Brain metastases	ACEIs/ARBs vs. non-users	32 vs. 79	11.6 vs. 15.3	−3.7
Maloney (2022)	Early-stage lung cancer	ARBs vs. non-users	24 vs. 223	96.7 vs. 43.3	53.4
**Recurrence-free survival time**					
Januel (2015)	Supratentorial glioblastoma	ACEIs/ARBs vs. non-users	26 vs. 55	8.8 vs. 7.4	1.4
Maloney (2022)	Early-stage lung cancer	ARBs vs. non-users	24 vs. 223	64.3 vs. 35	29.3
**Study**	**Cancer**	**Comparison**	**Event Rate**	**Odds Ratio**	**95%CI**
**Overall survival rate (2-year)**					
Harder (2015)	Primary lung cancer	ACEIs vs. non-users	42/70 vs. 99/187	1.33	0.76, 2.33
Harder (2015)	Primary lung cancer	ARBs vs. non-users	13/35 vs. 107/187	0.44	0.21, 0.93 *

**Total recurrence rate**					
Maloney (2022)	Early-stage lung cancer	ARBs vs. non-users	3/24 vs. 53/223	0.46	0.13, 1.60
Total death rate					
Maloney (2022)	Early-stage lung cancer	ARBs vs. non-users	5/24 vs. 124/223	0.21	0.08, 0.58 *
**Death due to disease progression rate**					
Wedlake (2012)	Pelvic malignancies	ACEIs vs. non-users	6/39 vs. 20/198	1.62	0.60, 4.33
**Death due to cancer-related treatment toxicity rate**					
Wedlake (2012)	Pelvic malignancies	ACEIs vs. non-users	3/39 vs. 7/198	2.27	0.56, 9.21

(Note) ACEIs: angiotensin-converting enzyme inhibitors. ARBs: angiotensin receptor blockers. CI: confidence interval. * significant difference.
